# Long-term impact of community-based participatory women’s groups on child and maternal mortality and child disability: follow-up of a cluster randomised trial in rural Nepal

**DOI:** 10.1136/bmjgh-2018-001024

**Published:** 2018-12-01

**Authors:** Michelle Heys, Lu Gram, Angie Wade, Edward James Norman Haworth, David Osrin, Khadkha Sagar, Dej Krishna Shrestha, Rishi Prasad Neupane, Dhruba Adhikari, Ramesh Kant Adhikari, Bharat Budhathoki, Dharma Manandhar, Anthony Costello

**Affiliations:** 1 UCL Institute for Global Health, University College London, London, UK; 2 Great Ormond Street Institute of Child Health, University College London, London, UK; 3 Mother and Infant Research Activities (MIRA), Kathmandu, Nepal; 4 Health Systems Unit, WHO Country Office for Nepal, Kathmandu, Nepal

**Keywords:** child mortality, maternal mortality, developmental disabilities, Nepal, observational study

## Abstract

**Background:**

Community-based women’s groups practising participatory learning and action (PLA) can reduce maternal and neonatal mortality in low-income countries. However, it is not clear whether these reductions are associated with subsequent increased or decreased rates of childhood death and disability. We assessed the impact on child deaths and disability beyond the perinatal period among participants in the earliest trial in Nepal 2001–2003.

**Methods:**

Household interviews were conducted with mothers or household heads. At cluster and individual levels, we analysed disability using pairwise log relative risks and survival using multilevel logistic models.

**Findings:**

From 6075 children and 6117 mothers alive at 4 weeks post partum, 44 419 children (73%) were available for interview a mean 11.5 years later. Rates of child deaths beyond the perinatal period were 36.6 and 52.0 per 1000 children in the intervention and control arms respectively. Rates of disability were 62.7 and 85.5 per 1000 children in the intervention and control arms respectively. Individual-level analysis, including random effects for cluster pairing and adjusted for baseline maternal literacy, socioeconomic status and maternal age, showed lower, statistically non-significant, odds of child deaths (OR 0.70 (95% CI 0.43 to 1.18) and disability (0.64 (0.39 to 1.06)) in the intervention arm.

**Conclusion:**

Community-level exposure to women’s groups practising PLA did not significantly impact childhood death or disability or death beyond the perinatal period. Follow-up of other trials with larger sample sizes is warranted in order to explore the possibility of potential long-term survival and disability benefits with greater precision.

**Key questions**

**What is already known?**

- There is increased emphasis through the United Nations Sustainable Development Goals on enabling children to thrive, both in terms of growth and development.
- Women’s groups practising participatory learning and action (PLA) are a cost-effective way of improving newborn and maternal survival. However, it is not clear whether these reductions are associated with increased or decreased rates of subsequent childhood disability.

**What are the new findings?**

- This study is the first ever long-term follow-up of a trial of community-based participatory women’s groups practising PLA.
- Community-level exposure to community-based women’s groups practising PLA did not significantly impact disability or death among children surviving the neonatal period.

**What do the new findings imply?**

- Our findings add impetus for rural low-income country settings to adhere to WHO guidance and roll out community-based participatory women’s groups in such areas.

## Introduction

Almost two-thirds of the world’s children (61%) live in low-middle or lower-middle-income countries (LIC and LMIC).^1^ Nepal is one of 31 LIC and one of just three LIC outside sub-Saharan Africa. Neonatal and under five mortality rates have fallen in LIC and LMIC over the past two decades but remain unacceptably high.^2^ The United Nations Sustainable Development Goals place renewed emphasis on broad-based interventions that produce lasting reductions in childhood mortality and on disability and inclusivity that allow all children to thrive. Limited data of good quality exist around the epidemiology of childhood disability in developing countries and particularly long-term developmental outcomes following key exposures and interventions in the perinatal period.^3 4^ It is therefore important to assess the impact of perinatal interventions on mortality and morbidity beyond the newborn period to ascertain whether such interventions not only save lives but reduce disability.

In 2001–2003, in Makwanpur, Nepal, a cluster randomised controlled trial (RCT) of community-based women’s groups practising participatory learning and action (PLA) reported improvements in newborn and maternal survival.^5^ The trial was conducted by Mother and Infant Research Activities (MIRA) in partnership with the Institute for Global Health, University College London, UK. The women’s groups, which were facilitated by a local woman (non-health professional), explored health issues around pregnancy, childbirth and newborn health through monthly meetings over 3 years, in other words for at least 2 years after the child was born. The trial showed a 30% reduction in newborn mortality, and a significant fall in maternal mortality although this was not a primary hypothesis. Data collected 12–18 months after trial completion showed evidence of sustainability, with 80% of groups still active.^6^ Similar trials were conducted in Bangladesh, India and Malawi. A meta-analysis showed that, with a minimum attendance by 30% of pregnant women, exposure to community women’s PLA groups led to a 33% reduction (95% CI: 25% to 40%) in neonatal mortality rate (NMR) and a 49% (11%–71%) reduction in maternal mortality rate.^7^ On the basis of this evidence, the WHO recommended the implementation of community mobilisation through women’s groups practicing PLA cycles to improve maternal and newborn health, especially in rural settings with poor access to healthcare.^8^

Despite evidence of efficacy, equity^9^ and cost-effectiveness,^7^ understanding is evolving of pathways to impact that are likely to be multifactoral and context specific.^5 10 11^ Potential mechanisms include biological, behavioural and psychosocial factors. Adequate community coverage seems to be key to successful implementation.^10^ Community-level exposure (to a lesser degree) and individual attendance (to a greater degree) at the groups appears to result in changes in home birth practices and careseeking behaviour for maternal health,^5 7 10^ leading to reductions in maternal infection rates and more timely intervention as required at delivery.^5 7^ Women attending the groups, at least in the short term, perceive them to have resulted in increased self-confidence and self-esteem, greater social support^12 13^ and increased participation in household decision-making, particularly around healthcare (although other studies have not shown impact on household decision making).^14 15^ Qualitative data suggest they can build solidarity, share resources, reduce stress and assist communities to lobby for better access to care and rights.^16^

Perinatal infection and obstructed delivery are risk factors for childhood disability and morbidity, and therefore potentially subsequent mortality beyond the perinatal period. For example, key risk factors for the most common physical disability in childhood, cerebral palsy,^17^ are neonatal infection and hypoxic-ischaemic encephalopathy.^18^ On the other hand, there is evidence that some perinatal interventions may result in reduced mortality but greater morbidity and disability. This was seen in the early years following improvements in newborn intensive care in high-income settings, when mortality for premature infants fell, but disability rates rose.^19^ Furthermore, the efficacy of some perinatal interventions appears to be context specific.^20^

The community-based women’s groups continued beyond the perinatal period, extending to cover “the first 1000 days”. It is widely recognised that this critical period—defined approximately by the time between conception and the child’s second birthday—presents a potentially modifiable set of exposures that affect the foundations of health, growth and cognitive development. It is possible, therefore, that exposure to women’s groups beyond the perinatal period may also have affected risk of survival and disability in offspring.

Our study aimed to examine survival and disability outcomes among 6075 children aged from 9·4 to 13·1 years of age enrolled in the cluster RCT of women’s groups in Makwanpur, Nepal. The long-term impact of community-based women’s groups practising a PLA approach to child survival and disability has not been explored. Would group activities have sustained effects on survival into childhood, through reduced perinatal morbidity (from, for example, neonatal sepsis) that might otherwise be associated with childhood mortality and disability, or through sustained changes in factors such as maternal healthcare-seeking behaviour that result in ongoing improvements in child health? Might increased neonatal survival be associated with more disability in surviving children, as vulnerable infants who would have otherwise died survive with impairment?

## Methods

### Setting

Nepal is a country of 28·7 million people with a gross per capita income of USD $1147 and a low Human Development Index.^21^ Makwanpur district is a rural hill area in central Nepal in which most households depend on subsistence agriculture (population >500 000 in 2014). Geographical details and cluster maps are in the trial paper.^5^

### Study design and participants

Our study was a follow-up observational survey of the trial cohort. We carried out face-to-face home interviews with children and mothers who were contactable and willing participants from the original trial cohort who had survived beyond 4 weeks post partum. We collected reported participant survival outcomes from the closest available family member, or from neighbours when families had moved and were untraceable. Twenty-four pairs of trained field interviewers and assistants were deployed to the 24 original trial clusters. Seven field supervisors observed 18% interviews to ensure data quality. Data were collected on android tablets using commcareHQ.^22^

### Data collection

Data were collected in two rounds with closed questionnaires. Mortality and disability data were gathered in the first round (January to July 2014). Other data collected included anthropometry, maternal reproductive history, literacy and age and measures of household socioeconomic status (assets, landholding, home and animal ownership) and occupation and household food sufficiency.

### Pilot testing

Research tools for round 1 were piloted with 531 mother–child pairs chosen from six clusters. These clusters were randomly selected and stratified by allocation (but not pairing). The first 100 mother–child pairs were then selected by birth from the six clusters (giving a potential 600 participants) for the pilot study. Analysis of pilot data and field interviewer feedback were used to revise the final questionnaire and provide additional field worker training on, for example, waist circumference measurement. Questions about survival and disability did not change substantially, and pilot data were analysed with the main study data.

### Outcomes

Survival data obtained from face-to-face interview with participants were termed reliable. Survival data obtained by proxy from closest family or neighbours were termed probable. There were two main outcomes*—*reliable child deaths and childhood disability—and one secondary outcome: estimated child deaths (the sum of reliable and probable child deaths). Here, we refer to child deaths as deaths in children who were alive at 4 weeks post partum (in otherwords, at trial completion), but who died before follow-up. For completeness, maternal mortality outcomes were also gathered and described.

Childhood disability was assessed using the Module on Child Functioning and Disability (MCFD) produced by Unicef and the Washington Group on disability statistics for use in children and young people aged 2 to 17 years.^23^ The MCFD tool builds on the established Short Set of Questions for adult disability screening.^24^ At the time of the study, the tool was in the final phases of validation. It was chosen because it is based on a well established, frequently used screening measure for disability in large studies, it had been used successfully in this setting before, and it will be used in future large-scale screening for childhood disability, thus allowing for future comparisons.

The child’s main caregiver was asked 19 questions to assess functioning across six core functional domains—speech and language, hearing, vision, learning, mobility and motor skills—and six extended domains: self-care, emotions, behaviour, attention, relationships and playing (online Appendix table A1). Responses were ranked and scored as no difficulty (1), some difficulty (2), a lot of difficulty (3) or cannot do at all (4). Owing to the current absence of validity data on extended questions, Unicef and the Washington Group on disability statistics have advised the definition of disability to be the report of at least some difficulty in at least one of the six core functional domains. We therefore defined a positive disability screen as at least some difficulty (score ≥2), in at least one core domain. This is the most inclusive definition of disability.^25^

10.1136/bmjgh-2018-001024.supp1 Supplementary data

### Potential confounders

Baseline differences between mothers in the intervention and control arms in socioeconomic status and maternal literacy were reported in the original trial and were considered as potential confounders here, collected using the same methods at trial and long-term follow-up. Additional confounders considered included gender of child, caste, primary household occupation, househood food sufficiency, maternal age and gender of household head. From these, potential additional confounders only those with significant differences between intervention and control arm were included in the final models.

### Possible confounding from subsequent implementation of community-based women’s groups in the trial area

Following trial completion, there were two potential periods of subsequent exposure to PLA groups for women and their families. First, from July 2005 to December 2008, the trial control clusters were offered the original PLA activities, and the trial intervention clusters were offered augmented PLA activities focusing on careseeking for childhood illness and additionally involving men. Second, from October 2010 to September 2012, the “Skilled Birth Attendant Trial” (SBA trial)^26^ was conducted in Makwanpur testing the impact on increasing SBA of combined PLA groups with strengthening of health management committees. During the SBA trial, and independent of previous randomisation, all 43 village development committees (VDCs) in Makwanpur district were randomised to intervention (n=21) or control (n=22). MIRA did not run PLA groups in the SBA control clusters. The curricula of the three models of PLA group meetings are outlined in online supplementary table 1.

We did not conduct detailed modelling of subsequent potential exposure to PLA groups in the years following the first trial completion for a number of reasons. First, we were unable to map attendance at women’s groups across the period and trials due to a lack of data and change in methodology in creation of participant unique identifiers. Second, if we assume our index mothers would not continually attend women’s groups unless they became pregnant again, our data suggest the percentage of women from the original trial closed cohort with subsequent pregnancies during future potential recruitment periods was low: 31% of the original cohort delivered again during the period when the control clusters were offered the original PLA, while only 2% delivered again during the SBA trial.^27^ Finally, we assume that subsequent exposure to women’s groups would have minimal impact on disability or mortality outcomes for our maturing index offspring given the perinatal focus of discussion topics in both the original trial and the SBA trial (online supplementary table A2).

### Statistical analysis

The analysis plan was conceived a priori and was devised to follow the analysis plan for the original trial. We performed intention-to-treat analyses for all outcomes. Those who moved between intervention and control clusters after recruitment to the original trial were analysed according to trial arm allocation at recruitment.

#### Cluster level

Cluster level analyses were performed using death or disability rate per 1000 population and relative risk (RR). Child death rates per 1000 person years per cluster were calculated using a person-years denominator calculated from age at follow-up or reported age of death (available for all but three children). Death and disability rates showed a skewed distribution. A Wilcoxon signed rank test was performed on non-transformed data and F test for difference in variance. The within-pair log RRs of death and disability rates (adding 0·5 to the counts to allow for zero event rates)^28^ were compared within regression models adjusting for differences in cluster sample sizes.

#### Individual level

Multilevel (two levels: cluster and cluster pairing) logistic regression analyses were performed to provide consistency with the original trial analysis. We estimated odds of death or disability in the intervention compared with the control group. We carried out adjusted analyses by calculating a propensity score and entering it into the main regression of trial outcomes on trial arm. We calculated the propensity score using a single level logistic regression of trial arm on maternal education at baseline and household asset score. We used propensity scoring because the estimation algorithm failed to converge in Stata^29^ when we directly entered education and household assets into the main regression. Adjustment using propensity scores is statistically equivalent to directly entering the control variables into a multivariable regression, but numerical computation of maximum likelihood estimates often succeeds with propensity scoring, where it fails with directly entered covariates due to the fewer number of variables involved in the main regression.^30^ Intraclass correlation coefficients (ICCs) were calculated for child and maternal deaths (from 4 weeks to follow-up) and disability, compared with those from the trial outcomes at 4 weeks.

Additional sensitivity analysis was performed using a crude Cox regression model with fixed stratum-level effects and shared frailty at the VDC level for time to death (reliable child deaths).

Statistical analyses were performed using R V.2·3·1^31^ and Stata V.14·0.^29^

## Results

**Figure 1:**
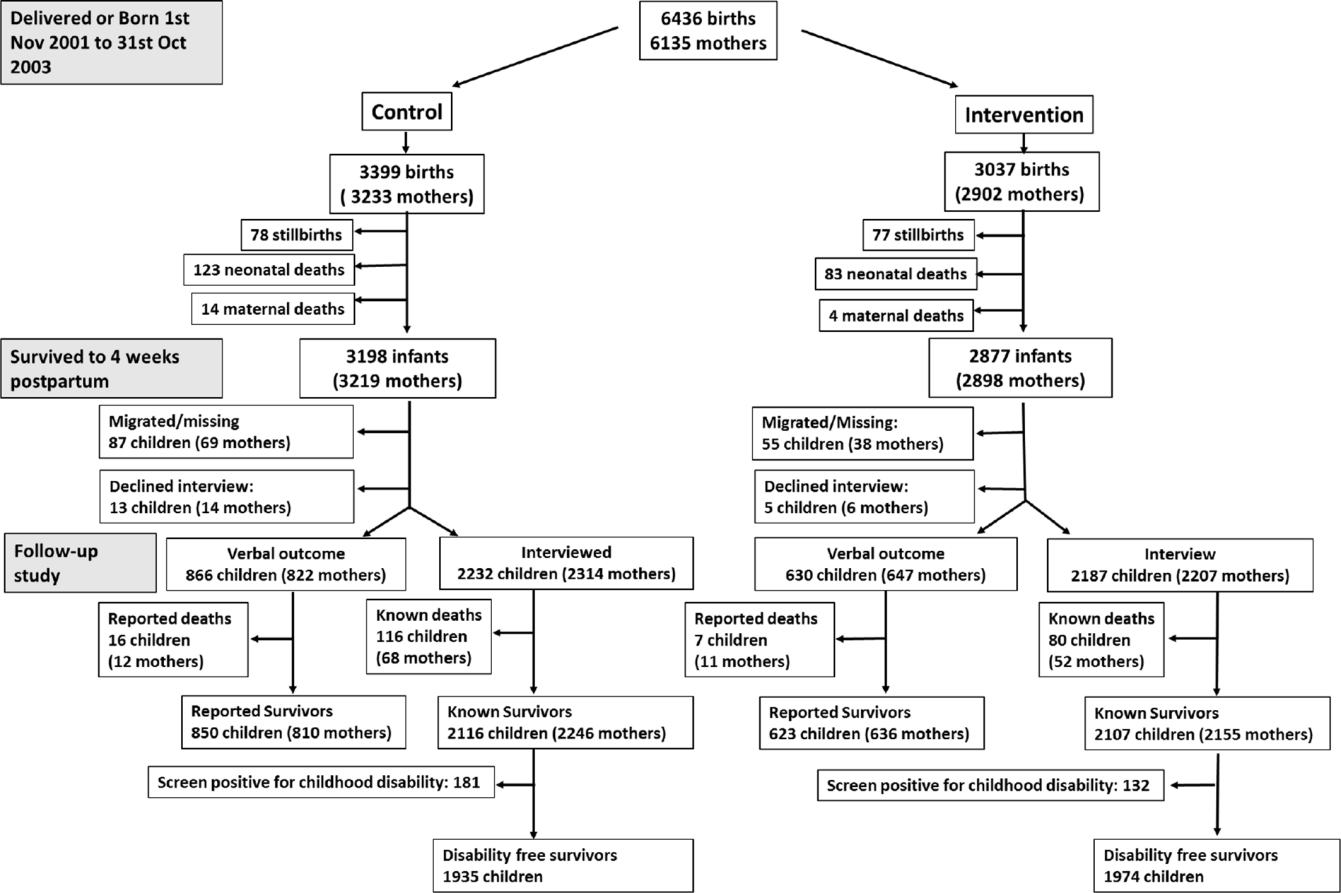
Trial and follow-up participant flow chart showing mortality and disability outcomes from recruitment to follow-up approximately 11.5 years later.

Figure 1 shows child and maternal survival, and the numbers of surviving children screening positive for disability, by trial allocation. The original trial analysed data from 6272 children and 6001 mothers. For completeness, data on an additional 164 infants and 134 mothers that were not available in the original trial analysis were included. About 6075 children and 6117 mothers survived to 4 weeks post partum. Of the 6075 surviving children, 18 (0·3%) declined interview, and no survival data at all were available for 142 missing or migrated children. Of the 6117 surviving mothers, 20 (0·3%) declined interview, and no survival data at all were available for 107 missing or migrated mothers. We therefore collected survival data on 5990 mothers and 5915 children. Of these, 4419 children (73%) and 4521 (74%) mothers were available for interview. An additional 1496 children (25%) and 1469 (24%) mothers had relatives or neighbours who were able to provide information on survival. We collected disability outcomes on 4222 children.

Children were a mean age of 11·5 years (SD 0·6, range 9·4–13·1) and mothers were 38·8 years (6·7, 24–64 at follow-up. Of the 196 children who were reported as having died after 4 weeks of age, 193 families were able to provide an approximate age of death (mean 2·4 years (SD 2·8, 0·1–11). The overall prevalence of disability using the definition of at least some difficulty in at least one core domain was 7·4%, with 6·0% of children screening positive for physical disability, 2·7% for learning disability and 2·3% for behavioural disability. Prevalence of disability using the cut-off of a lot of difficulty in at least one core domain was 1·0%.

Table 1 shows baseline characteristics of children who were interviewed compared with those who were not by trial allocation status. Children who were interviewed had similar baseline socioeconomic characteristics and maternal literacy status at birth compared with children not interviewed, but children unavailable for interview were more likely to have been allocated to the control intervention arm. Children who were allocated to the intervention arm, irrespective of availability for follow-up interview, were more likely to live in households where the primary occupation was agriculturally related. Children who were allocated to the intervention arm and were followed-up were more likely to have older mothers.

**Table 1:** Baseline characteristics at follow-up for children interviewed at long-term follow-up compared with those not interviewed by trial allocation (including those who declined participation, but excluding those who died before 4 weeks of age)

	Available for interview		Not available for interview		Total count
	Exposure to the original Makwanpur trial		Exposure to the original Makwanpur trial		
	Intervention	Control	Intervention	Control	
Total number of children	51% (2232)	49% (2187)	58% (966)	42% (690)	6075
Sex of child					
Female	49% (1086)	48% (1052)	45% (436)	48% (328)	48% (2902)
Male	51% (1143)	52% (1130)	55% (525)	52% (354)	52% (3152)
Caste					
Janajati	82% (1827)	74% (1616)	80% (769)	68% (462)	77% (4674)
Newar	2% (34)	3% (59)	3% (25)	2% (14)	2% (132)
Brahmin/Chhetri	12% (261)	18% (400)	13% (122)	25% (169)	16% (952)
Dalit	4% (87)	4% (97)	4% (38)	4% (28)	4% (250)
Other	1% (20)	0% (10)	1% (7)	1% (9)	1% (46)
Primary household occupation					
Agriculture	95% (2121)	87% (1902)	95% (917)	87% (591)	91% (5531)
Waged labour	3% (71)	8% (178)	2% (19)	8% (57)	5% (325)
Salaried/government job	1% (16)	3% (63)	1% (14)	3% (20)	2% (113)
Small business	1% (21)	2% (39)	1% (11)	2% (14)	1% (85)
Asset score					
None	56% (1251)	51% (1103)	55% (532)	52% (353)	54% (3239)
Clock, radio, iron or bicycle	35% (781)	31% (685)	36% (345)	33% (227)	34% (2038)
More costly assets	9% (197)	18% (394)	9% (84)	15% (102)	13% (777)
Household food sufficiency annually					
Fewer than 8 months	28% (635)	31% (684)	31% (296)	35% (236)	31% (1851)
More than 8 months	72% (1594)	69% (1498)	69% (665)	65% (446)	69% (4203)
Maternal education					
No education	87% (1937)	75% (1637)	84% (812)	72% (494)	81% (4880)
1–3 years	5% (101)	8% (178)	4% (42)	7% (46)	6% (367)
4–8 years	7% (160)	14% (307)	9% (87)	18% (121)	11% (675)
9+ years	1% (31)	3% (60)	2% (20)	3% (21)	2% (132)
Maternal age					
<25 years old	40% (886)	45% (983)	47% (451)	48% (326)	44% (2646)
25–29 years old	26% (579)	26% (558)	22% (213)	27% (183)	25% (1533)
30+ years old	34% (764)	29% (641)	31% (297)	25% (173)	31% (1875)
Gender of household head					
Male	95% (2117)	91% (1991)	96% (920)	91% (618)	93% (5646)
Female	5% (112)	9% (191)	4% (41)	9% (64)	7% (408)
Missing values (n)					
	3	5	5	8	21

Table 2 shows summary measures for the two treatment arms with death rate differences and rate ratios shown by individual. Fewer children and mothers died after 4 weeks post partum in the intervention arm than in the control arm and fewer children who survived screened positive for disability in the intervention arm. Absolute numbers of deaths and disability rates and risk by cluster pair at interview are in online appemdix tables 2A, 2B, 2C. There was no difference in cluster variance of risk of child or maternal deaths, or child disability, by trial allocation status (F-test statistics all >0·98, p values all >0·49). Wilcoxon signed rank tests of death and disability rates and RR of death or disability were all non-significant for all outcomes (p values all >0·17).

**Table 2:** Individual-level summary data for death and disability rates by treatment arm and estimated intervention effects

Reliable child deaths per 1000 children			
	Control	Intervention	
Total deaths	116	80	
Total children followed-up	2232	2187	
Analysis based on individual-level data			
Overall rate/1000 children	52·0	36·6	
Rate difference (per 1000 children)			−15·4 (95% CI −3.2 to 27.7)
Percentage difference in rate (%)			−29·6
Rate ratio			0·70

Reliablechilddeaths per1000 person-years			
	Control arm	Intervention arm	
Total deaths	115*	78*	
Total person-years	24424·3	24323·5	
Analysis based on individual-level data			
Overall rate/1000 person-years	4·7	3·2	
Rate difference (per 1000 person-years)			−1·5
Percentage difference in rate (%)			−31·9
Rate ratio			0·68

Reliable maternal deaths per 1000 women			
	Control arm	Intervention arm	
Total deaths	68	52	
Total women followed-up	2314	2207	
Analysis based on individual-level data			
Overall rate/1000 women	29·4	23·6	
Rate difference (per 1000 women)			−5·8
Percentage difference in rate (%)			−19·8
Rate ratio			0·80

Child disability per 1000 children interviewed			
	﻿Control arm	Intervention arm	
Total disability screen positive	181	132	
Total children followed-up	2116	2106†	
Analysis based on individual-level data			
Overall rate/1000 children	85·5	62·7	
Rate difference (per 1000 children)			−22·9 (95% CI -38.87 to 7.0)
Percentage difference in rate (%)			−26·7
Rate ratio			0·73

*Excludes three child deaths with missing data on age of death.

†Excludes one child with missing data on disability.

Table 3 shows the summary RRs (95% CI) weighted according to population size within clusters. There was a non-significant reduction in risk of reliable and estimated child and maternal death and childhood disability. For child deaths, considering deaths per 1000 person-years and weighting according to person-years of follow-up gave similar results (RR=0·65 (0·37 to 1·14), p=0·16).

**Table 3:** Relative risks (RRs) weighted according to population size within clusters

	Cluster level analyses weighted by cluster size	
	RR (95% CI)	P values
Reliable child deaths	0·73 (0·42 to 1·27)	0·24
Estimated child deaths	0·72 (0·43 to 1·22)	0·20
Child disability	0·69 (0·41 to 1·16)	0·15
Reliable maternal deaths	0·78 (0·48 to 1·28)	0·30
Estimated maternal deaths	0·84 (0·54 to 1·32)	0·42

CI: confidence interval; RR: relative risk

Table 4 shows the results of the multilevel analysis of individual data for children and mothers. In the model with a fixed effect for cluster pairing, but not for baseline asset score and maternal literacy, children born in intervention clusters had 30% lower odds of (reliable) deaths after 4 weeks of age and up to age of follow-up compared with children born in control clusters (OR 0·70, 95% CI 0·50 to 0·97). Additional adjustment for baseline asset score and maternal literacy or inclusion of a random effect for cluster pairing revealed similar but non-significant effect sizes. Adjusted with fixed effects for cluster pairing and unadjusted or additionally adjusted for baseline asset score and maternal literacy, children also had a 34% lower odds of disability at mean age 11·5 years compared with children born into control clusters (OR 0·66 (0·46 to 0·94)). Again, estimates were similar, but non-significant when models included a random effect for cluster pairing. All individual-level analyses revealed a non-significant reduction in (reliable and estimated) maternal ﻿deaths.

Results were robust to sensitivity testing using a crude Cox regression model with fixed stratum-level effects and shared frailty at the VDC level (data not shown).

**Table 4:** Unadjusted and adjusted individual-level analysis showing odds of reliable and estimated child and maternal deaths and disability

			Control	Intervention	Random stratum effects		Fixed stratum effects	
					Unadjusted	Adjusted	Unadjusted	Adjusted
					OR (p value; 95% CI)	OR (p value; 95% CI)	OR (p value; 95% CI)	OR (p value; 95% CI)
Child deaths*			n	n				
Follow-up data only	Reliable	Died	116	80				
		Alive	2116	2107	0·67 (0·15; 0·39 to 1·16)	0·70 (0·18; 0·43 to 1·18)	0·70 (0·034; 0·50 to 0·97)	0·76 (0·075; 0·55 to 1·03)
	Total estimated	Died	132	87				
		Alive	2966	2730	0·71 (0·15; 0·44 to 1·14)	0·75 (0·17; 0·50 to 1·14)	0·68 (0·007; 0·51 to 0·90)	0·72 (0·026; 0·54 to 0·96)
**Maternal deaths (per individual mother**)								
Follow-up data only	Reliable	Died	68	52				
		Alive	2246	2155	0·75 (0·26; 0·48 to 1·17)	0·83 (0·42; 0·53 to 1·31)	0·79 (0·28;0·54 to 1·15)	0·87 (0·47; 0·61 to 1·26)
	Total estimated	Died	80	63				
		Alive	3056	2791	0·83 (0·45; 0·56 to 1·24)	0·91 (0·67; 0·61 to 1·38)	0·85 (0·44; 0·61 to 1·20)	0·93 (0·69; 0·67 to 1·31)
**Child disability**								
	Screened	Positive	181	132				
		Negative	1935	1974	0·66 (0·10; 0·40 to 1·09)	0·64 (0·085; 0·39 to 1·06)	0·65 (0·021; 0·46 to 0·94)	0·63 (0·013; 0·44 to 0·91)

All models take account of clustering of women within village development committees and also the pairing of clusters (strata) using either ‘random stratum effects’ or ‘fixed stratum effects’. Adjusted OR are adjusted for baseline asset score, maternal literacy, maternal age and main household occupation using propensity scoring.

Child death rate excludes stillbirths and, and deaths in infants up to 4 weeks of age.

Table 5 shows ICCs for the main outcomes at follow-up compared with those at baseline.

**Table 5:** ICCs for mortality and disability outcomes

	Outcome	n	ICC
10–13 years follow-up	Childhood deaths	4419	0·02075
	Maternal deaths (per pregnancy)	4761	0·00402
	Maternal deaths (per individual mother)	4521	0·00220
	Childhood disability	4222	0·02140
4-week follow-up	Neonatal mortality	6436	0·00090
	Maternal mortality (per pregnancy)	6436	0·00027

ICC: Intra-class correlation coefficients (using standard ANOVA model)

## Discussion

This was the first ever long-term follow-up of a trial of community-based participatory women’s groups practising PLA. Among survivors beyond 4 weeks after birth, around 30% fewer children died in intervention than in control arm 27% fewer surviving children screened positive for disability. These reductions were significant only in models including fixed effects for cluster pairing and can therefore be viewed only as suggestive but not conclusive of ongoing survival benefits and reduction in childhood disability. Findings from the models accounting for cluster pairing have greater generalisability. Of key import is that community-based women’s groups practising PLA did not significantly impact disability or death among children surviving the neonatal period, a hitherto unreported finding.

Our findings are biologically plausible, although we lack data with which to clearly describe mechanisms. As postulated a priori, exposure to the womens’ groups in the perinatal period could, through reduction in or better management of prenatal and perinatal risk factors such as prolonged labour and maternal sepsis, have resulted in reduced rates of disability in babies who survived and who would have otherwise had associated disability. Alternatively, or simultaneously to a greater or lesser extent, the same mechanisms could have resulted in survival of babies who might have otherwise have died and who were at greater risk of disability. Additionally, ongoing exposure to womens’ groups during the critical first 1000 days could have further improved neurocognitive outcomes for offspring.

An additional finding was that 30% fewer mothers died after the perinatal period in intervention than in control arms. This was not significant in any model and should be considered an exploratory finding only. It is less clear why exposure to the perinatal groups should be associated with better long-term survival in mothers.

Generally, follow-up of perinatal intervention studies in low-income settings is unusual beyond the neonatal period, with no assessments of sustained impact on mortality beyond infancy and limited evidence about longer-term development and disability. A review of the literature revealed only three studies reporting outcomes of a perinatal intervention beyond infancy and early childhood in under-resourced settings, all reporting outcomes of prenatal micronutrient supplements in Nepalese children.^32 33^ A number of other studies have reported outcomes in early childhood. In Bangladesh, an RCT of prenatal food and micronutrient supplementation has been followed-up to 5 years, demonstrating both favourable and unfavourable effects.^34^ An international RCT of infants with birth asphyxia in LIC showed that early intervention with home therapy visits from 2 weeks of age made a small improvement to cognitive development scores at 3 years of age.^35^ In India, an observational study on the impact of home-based neonatal care^36^ and a cluster RCT (cRCT) on the use of community health workers to deliver postnatal interventions showed that reductions in NMR were sustained at 1 year, but without significant further impact on infant mortality rate.^37^ In Malawi, a multifactorial cRCT of perinatal participatory women’s groups and peer counsellors collected mortality data to 1 year and showed that both interventions decreased mortality, but analysis was complicated by interactions between the two interventions.^38^ In rural China, a RCT found a small improvement in mental development scores at 12 months in infants whose mothers were given multiple micronutrients during pregnancy compared with iron and folic acid alone.^39^

The strengths of the study include its uniqueness and high retention at follow-up for interview (73%). This is good for any cohort. No original plan was made to interview participants beyond trial completion, and the geographical and logistical difficulties of the study setting are formidable. Furthermore, we were able to obtain reported survival outcomes for an additional 24% of the cohort, due to limited internal migration and community cohesion. Additional strengths include the use of a broad measure of disability and adjustment for baseline differences in socioeconomic status.

The major limitation of the study was a relative lack of statistical power with which to determine long-term survival outcomes. The size of the study was determined by the original trial, and the sample size calculations for the follow-up study indicated that it would be possible to detect a difference of 5% in disability rates with 80% power assuming a baseline rate in the controls of 27% with a sample size of 3999. In these a priori sample size caluculations, α was set at 0.00833 to adjust for two-tailed comparisons (ie, allowing for the possibility of either group—intervention or control—having higher prevalence of childhood disability) and for multiple testing of 3 primary outcomes between intervention versus control arms. We assumed a conservative estimate of coefficient of variation (*k*) of 0.16 which was estimated using unpublished data on ICC from a study of maternal disability in Nepal from our group and from assumptions based on characteristics of clusters that would account for some of the variation in disability scoring between clusters. In fact, the ICCs for survival and disability outcomes were considerably higher than the original study and higher than we predicted, suggesting substantial intercluster variability. The observed prevalence of disability was much lower than predicted. This baseline rate of 27% was based on the only available study at the time reporting disability prevalence in children in rural Nepal.^40^ Wu *et al* reported on children aged 1–9 years with mean age 5 years, where disability was defined as a positive response to one of the items of the Ten Questions Questionnaire.^40^ The lower prevalence in our population may have been due to differences in demographic characteristics and/or age of the children. Wu *et al* report outcomes from Sarlahi district in Southern Nepal. Twenty per cent of the mothers in their study were illiterate (compared with 30% in our cohort). Nepal household census data from 2011 reports that per capita income in Makwanpur district is 25% above average, where as in Sarlahi district it is two-thirds the national average. Finally, the original trial contained relatively small numbers of clusters per arm (n=12), and individual analysis may be less robust.^28^ The absolute baseline difference (table 2) observed was 2.29% in favour of the intervention group (lower confidence limit 0.7%, upper confidence limit 3.9%). The adjusted difference in OR for disability (table 4, model) showed a 36% reduction in odds of childhood disability in favour of the intervention group (lower confidence limit of 61% reduction or upper limit of 6% increase in odds). The confidence limits give the precision of the estimates and show that we can reasonably discount the intervention group being associated with any clinically meaningful increase in disability.

Second, it is possible that residual confounding is a factor although randomisation should have reduced this likelihood; for example, if there were an exposure that was related to the outcome such as quality of water supply, for which data were not collected and which by chance was not equally distributed between the intervention and control arm. Third, deaths were recorded through verbal report because of weak vital registration. Fourth, we used a new disability screening tool, not yet validated in this population, but based on a validated screening tool (the Ten Questions Questionnaire).^24^ It was felt to be the best tool currently available. Finally, as the intervention was rolled out after trial completion and a second trial was conducted in the region, it is possible that control clusters also received benefit from the community-based women’s groups. This might have diluted any differential effect between intervention and control arm and attenuated our findings.

Future research should focus on follow-up of similar trials with larger sample sizes and greater numbers of clusters in order to confirm or refute our findings. A clinical validation study of the disability screening tool should also be conducted in this setting—particularly in view of the low prevalence rates using the more typical cut-off of “a lot of difficulty” in at least one domain.

## Conclusion

A low cost, community-based intervention shown to reduce the odds of neonatal and maternal mortality was not associated with later increased or decreased risk of subsequent childhood disability or death. Apparent reductions in childhood disability and deaths in the intervention arm did not reach statistical significance, possibly due to the study being underpowered to detect these long-term outcomes. The possibility of longer-term positive impact on survival and disability should be tested in a follow-up of similar, larger scale trials of community-based participatory women’s groups practising PLA. The policy implications of our findings, if confirmed, are significant. Women’s groups have broader benefits than information sharing. They can build solidarity. Our findings add impetus for rural LIC settings to adhere to WHO guidance and roll out community-based participatory women’s groups in such areas. They also highlight the importance of the perinatal period as a critical time to address maternal child health issues and set the path for healthier childhood.
