# Supplementary material for: Long-term impact of community-based participatory women’s groups on child and maternal mortality and child disability: follow-up of a cluster randomised trial in rural Nepal
Source: BMJ Glob Health. 2018 Dec 1;3(6):e001024. doi: 10.1136/bmjgh-2018-001024 (PMC6278922; doi:10.1136/bmjgh-2018-001024)
Supplement: Supplementary data [file bmjgh-2018-001024supp001.pdf]

Table A1: Module on child functioning and disability

Seeing	Children aged 2-17	Does [name] wear glasses or contact lenses?	1= Yes<br>0 =No
		[*if wears glasses*]<br>Does [name] have difficulty seeing, when wearing his/her glasses? Would you say…	1=No difficulty<br>2=Some difficulty<br>3=A lot of difficulty<br>4=Cannot do at all
		[*If child does NOT wear glasses*] Does [name] have difficulty seeing?	1=No difficulty<br>2=Some difficulty<br>3=A lot of difficulty<br>4=Cannot do at all
Hearing	Children aged 2-17	Does [name] use a hearing aid?	1= Yes<br>0 =No
		[*If child uses a hearing aid*] Does [name] have difficulty hearing, when using his/her hearing aid(s)?	1=No difficulty<br>2=Some difficulty<br>3=A lot of difficulty<br>4=Cannot do at all
		[*If child does NOT use a hearing aid*]<br>Does [name] have difficulty hearing?	1=No difficulty<br>2=Some difficulty<br>3=A lot of difficulty<br>4=Cannot do at all
Walking	Children aged 5-17	Compared with children of the same age, does [name] have difficulty walking 500 yards/meters on level ground? That would be about the length of 5 football fields.	1=No difficulty<br>2=Some difficulty<br>3=A lot of difficulty<br>4=Cannot do at all
		Compared with children of the same age, does [name] have difficulty walking 100 yards/meters on level ground? That would be about the length of 1 football field.	1=No difficulty<br>2=Some difficulty<br>3=A lot of difficulty<br>4=Cannot do at all
Self-care	Children aged 5-17	Compared with children of the same age, does [name] have difficulty with self-care such as feeding or dressing him/herself?	1=No difficulty<br>2=Some difficulty<br>3=A lot of difficulty<br>4=Cannot do at all
Communication and comprehension	Children aged 5-17	Compared with children of the same age and using [his/her] usual language, does [name] have difficulty understanding other people?	1=No difficulty<br>2=Some difficulty<br>3=A lot of difficulty<br>4=Cannot do at all
		Compared with children of the same age and using [his/her] usual language, does[name] have difficulty being understood by other people?	1=No difficulty<br>2=Some difficulty<br>3=A lot of difficulty<br>4=Cannot do at all
Learning	Children aged 3-17 years	Compared with children of the same age, does [name] have difficulty learning to do new things?	1=No difficulty<br>2=Some difficulty<br>3=A lot of difficulty<br>4=Cannot do at all
	Children aged 5-17	Compared with children of the same age, does [name] have difficulty remembering things that they have learned?	1=No difficulty<br>2=Some difficulty<br>3=A lot of difficulty<br>4=Cannot do at all
Emotions	Children aged 5-17	Compared with children of the same age, how much does [he /she] worry or feel sad? Would you say… [*Read response categories*]	1=The same or less<br>2=More<br>3=A lot more
Behaviour	Children aged 5-17	Compared with children of the same age, how much difficulty does [name] have controlling[his/her] behaviour?	1=No difficulty<br>2=Some difficulty<br>3=A lot of difficulty<br>4=Cannot do at all

Attention	Children aged 5-17	Compared with children of the same age, does [name] have difficulty completing a task?	1=No difficulty<br>2=Some difficulty<br>3=A lot of difficulty<br>4=Cannot do at all
Coping with change	Children aged 5-17	Compared with children of the same age, does [name] have difficulty accepting change to plans or routine?	1=No difficulty<br>2=Some difficulty<br>3=A lot of difficulty<br>4=Cannot do at all
Relationships	Children aged 5-17	Does [name] have difficulty getting along with children of [his/her] age?	1=No difficulty<br>2=Some difficulty<br>3=A lot of difficulty<br>4=Cannot do at all
Playing	Children aged 2-12 years	Compared with children of the same age, does [name] have difficulty playing with other children?	1=No difficulty<br>2=Some difficulty<br>3=A lot of difficulty<br>4=Cannot do at all

Table 1B. Activity schedule for all participatory women's group meetings 2001-2014.

Dates	*Nov 2001 - Jun 2005 for Areas 1 and 2*<br>*Jul 2005 - Dec 2008 for Areas 3 and 4*<br>*Jul 2006 - Dec 2008 for Areas 5 and 6*
**Meeting**	**Topic**
1	To introduce the study to the group
2	To discuss why mothers and newborn infants die and how the intervention will work in the community
3	To ascertain how women understand maternal and neonatal problems
4	To find out about maternal and neonatal problems in the community
5	To understand the frequency of maternal and neonatal problems and to identify strategies to obtain information in the community
6	To share information from other women in the community and to prioritise three important maternal and neonatal health problems
7	To discuss possible strategies for addressing the priority problems
8	To discuss involvement of other community members in developing strategies
9	To discuss preparation for a meeting of community members
10	To hold a meeting involving other community members to discuss the activities of the women's groups, the priority problems identified by the groups, and possible strategies, and reach consensus
Dates	*Jul 2005 - Dec 2007 for Areas 1 and 2*
**Meeting**	**Topic**
1	Introduce the new topic (caring for childhood illness) and how we plan to work
2	Plan how to inform the community of the new focus on child health, and who to invite<br>Plan how to continue existing work with newborn and maternal health
3	Introduce new members to the work of the group and the topic of child health and discuss the reasons why children die
4	To discuss illnesses that affect children, to explore reasons why they have this illness and to explore what action is taken when children have these illnesses
5	To discuss what criteria will be used in prioritising problems and to discuss how to collect data useful for prioritisation
6	To share information collected and to prioritise problems to address through strategies
7	To identify local resources available, and discuss ways to overcome prioritised problems<br>To discuss if health personnel should be invited to the meeting
8	To learn about medical causes and medical treatments for prioritised problems<br>To discuss strategies with health personnel (if they are available)
9	To discuss how illness are obtained and passed using different social games
10	Discuss the feasibility of strategies and how to disseminate strategies discussed
11	To share information with the community and plan how to implement the strategies

12	To discuss how the group will know if the strategies have been effective or not To plan how these strategies will be evaluated

Dates	*Jan 2008 - Jun 2008 for Areas 1 and 2*
**Meeting**	**Topic**
1	To discuss family decision-making in the ward and to discuss men's role in maternal and newborn care
2	To discuss barriers preventing supportive behaviour of men and to plan how to work with men to overcome these barriers
3	To plan how to work with men, talk to men, invite men to participate and evaluate the effectiveness of the chosen strategy
4	The women's group and local men meet to discuss their agenda and plan how to work together in the future

Dates	*Oct 2010 - Sep 2012 for Areas 2, 4 and 6*
**Meeting**	**Topic**
1	Introducing MIRA, working in groups and the importance of institutional delivery
2	Discussing community barriers to institutional delivery and how they can be overcome in general
3	Sharing personal experiences and information about community barriers to institutional delivery
4	Prioritising barriers to address through participatory voting and discussing strategies going forward
5	Preparing a community meeting to present barriers to institutional delivery and ways to address them
6	Holding a community meeting about addressing barriers to institutional delivery
7	Discussing the implementation of strategies and plan cluster-level interaction with other groups
8	Carrying out a cluster-level interaction with other groups in the community
9	Carrying out an internal evaluation of the work of the group
10	Planning an evaluation of the strategies the group has carried out
11	Presenting the results from the evaluation to the community
12	Planning how to improve existing strategies

Table 2A: Reported child mortality, maternal mortality and child disability absolute numbers, rates and risk by cluster pair at interview (so called, *reliable* mortality outcomes)

		Control			Intervention			Intervention versus control	
	cluster pair	deaths	cluster size	deaths per 1000 children	deaths	cluster size	deaths per 1000 children	RR mortality	RR+ mortality
Reliable Child Mortality	1	7	233	30	12	366	32.8	1.09	1.06
	2	14	201	69.7	1	197	5.1	0.07	0.11
	3	0	85	0	1	131	7.6	-	1.95
	4	8	116	69	5	115	43.5	0.63	0.65
	5	0	41	0	11	121	90.9	-	7.92
	6	11	226	48.7	6	178	33.7	0.69	0.72
	7	2	71	28.2	3	134	22.4	0.79	0.75
	8	14	494	28.3	8	318	25.2	0.89	0.91
	9	18	282	63.8	2	179	11.2	0.18	0.21
	10	4	85	47.1	7	156	44.9	0.95	0.91
	11	24	213	112.7	22	159	138.4	1.23	1.23
	12	14	171	81.9	2	132	15.2	0.19	0.22
	cluster pair	deaths	cluster size	deaths per 1000 pregnancies	deaths	cluster size	deaths per 1000 pregnancies	RR mortality	RR+ mortality
Reliable Maternal mortality	1	7	248	28.2	15	377	39.8	1.41	1.36
	2	5	217	23	5	205	24.4	1.06	1.06
	3	4	95	42.1	6	153	39.2	0.93	0.9
	4	7	149	47	4	128	31.3	0.67	0.7
	5	0	48	0	1	132	7.6	-	1.1
	6	12	236	50.8	4	184	21.7	0.43	0.46
	7	1	83	12	3	132	22.7	1.89	1.47
	8	11	568	19.4	7	318	22	1.14	1.16
	9	16	289	55.4	2	197	10.2	0.18	0.22
	10	6	93	64.5	0	163	0	-	0.04
	11	11	232	47.4	11	200	55	1.16	1.16
	12	5	185	27	2	129	15.5	0.57	0.65
	cluster pair	positive disability screen	cluster size	positive disability screen per 1000 children	positive disability screen	cluster size	positive disability screen per 1000 children	RR disability, Intervention versus control	
Child disability	1	12	226	53.1	26	354	73.4	1.38	
	2	14	187	74.9	26	197	132	1.76	
	3	10	85	117.6	6	130	46.2	0.39	
	4	5	108	46.3	17	110	154.5	3.34	
	5	4	41	97.6	13	110	118.2	1.21	
	6	16	215	74.4	10	172	58.1	0.78	
	7	5	69	72.5	1	131	7.6	0.11	
	8	42	480	87.5	8	310	25.8	0.29	
	9	10	264	37.9	4	177	22.6	0.6	
	10	11	81	135.8	8	148	54.1	0.4	
	11	24	189	127	5	137	36.5	0.29	
	12	28	171	163.7	8	130	61.5	0.38	

Table 2B: Reported child mortality, maternal mortality and child disability absolute numbers, rates and risk by cluster pair at interview (so called, *reliable* mortality outcomes)

		Intervention			Control			Intervention versus control	
	cluster pair	deaths	cluster size	deaths per 1000 children	deaths	cluster size	deaths per 1000 children	RR mortality	RR+ mortality
Section 1: Reliable Child Mortality	1	12	366	32.8	7	233	30	1.09	1.06
	2	1	197	5.1	14	201	69.7	0.07	0.11
	3	1	131	7.6	0	85	0	-	1.95
	4	5	115	43.5	8	116	69	0.63	0.65
	5	11	121	90.9	0	41	0	-	7.92
	6	6	178	33.7	11	226	48.7	0.69	0.72
	7	3	134	22.4	2	71	28.2	0.79	0.75
	8	8	318	25.2	14	494	28.3	0.89	0.91
	9	2	179	11.2	18	282	63.8	0.18	0.21
	10	7	156	44.9	4	85	47.1	0.95	0.91
	11	22	159	138.4	24	213	112.7	1.23	1.23
	12	2	132	15.2	14	171	81.9	0.19	0.22
	cluster pair	deaths	cluster size	deaths per 1000 pregnancies	deaths	cluster size	deaths per 1000 pregnancies	RR mortality	RR+ mortality
Section 2: Reliable Maternal mortality	1	15	377	39.8	7	248	28.2	1.41	1.36
	2	5	205	24.4	5	217	23	1.06	1.06
	3	6	153	39.2	4	95	42.1	0.93	0.9
	4	4	128	31.3	7	149	47	0.67	0.7
	5	1	132	7.6	0	48	0	-	1.1
	6	4	184	21.7	12	236	50.8	0.43	0.46
	7	3	132	22.7	1	83	12	1.89	1.47
	8	7	318	22	11	568	19.4	1.14	1.16
	9	2	197	10.2	16	289	55.4	0.18	0.22
	10	0	163	0	6	93	64.5	-	0.04
	11	11	200	55	11	232	47.4	1.16	1.16
	12	2	129	15.5	5	185	27	0.57	0.65
	cluster pair	positive disability screen	cluster size	positive disability screen per 1000 children	positive disability screen	cluster size	positive disability screen per 1000 children	RR disability, Intervention versus control	
Section 3: Child disability	1	26	354	73.4	12	226	53.1	1.38	
	2	26	197	132	14	187	74.9	1.76	
	3	6	130	46.2	10	85	117.6	0.39	
	4	17	110	154.5	5	108	46.3	3.34	
	5	13	110	118.2	4	41	97.6	1.21	
	6	10	172	58.1	16	215	74.4	0.78	
	7	1	131	7.6	5	69	72.5	0.11	
	8	8	310	25.8	42	480	87.5	0.29	
	9	4	177	22.6	10	264	37.9	0.6	
	10	8	148	54.1	11	81	135.8	0.4	
	11	5	137	36.5	24	189	127	0.29	
	12	8	130	61.5	28	171	163.7	0.38	

RR: relative risk; RR+: relative risk of mortality of children in intervention versus control where all events have been adjusted by adding 0.5 to event total

Table 2C: Reliable child mortality rates per 1000 person years observed among children from 4 weeks of age to time of follow-up in the 24 clusters of the cRCT

	Control			Intervention			Intervention versus control	
Cluster pair	deaths	person-years	rate/1000 person years	deaths	person-years	rate/ 1000 person years	RR mortality	RR+ mortality
1	7	2585.3	2.7	12	4128	2.9	1.07	1.06
2	14	2168.9	6.5	1	2245.2	0.4	0.06	0.11
3	0	975.2	0	1	1506.8	0.7	-	1.95
4	8	1253.7	6.4	5	1270.7	3.9	0.61	0.64
5	0	460.5	0	11	1285.5	8.6	-	8.24
6	11	2483	4.4	6	1968	3	0.68	0.71
7	2	797.7	2.5	2	1506.2	1.3	0.52	0.53
8	13	5532.4	2.3	8	3586.3	2.2	0.96	0.97
9	18	3024.5	6	2	2031.8	1	0.17	0.2
10	4	930.4	4.3	7	1714.4	4.1	0.95	0.9
11	24	2214.8	10.8	21	1592	13.2	1.22	1.22
12	14	1998	7	2	1488.7	1.3	0.19	0.23

RR: relative risk; RR+: relative risk of mortality of children in intervention versus control where all events have been adjusted by adding 0.5 to event total
